# Basic and supplementary sensory feedback in handwriting

**DOI:** 10.3389/fpsyg.2015.00169

**Published:** 2015-02-20

**Authors:** Jérémy Danna, Jean-Luc Velay

**Affiliations:** Aix Marseille Université, CNRS, LNC UMR 7291, MarseilleFrance

**Keywords:** handwriting, sensory feedback, vision, proprioception, audition, sonification, enriched reality

## Abstract

The mastering of handwriting is so essential in our society that it is important to try to find new methods for facilitating its learning and rehabilitation. The ability to control the graphic movements clearly impacts on the quality of the writing. This control allows both the programming of letter formation before movement execution and the online adjustments during execution, thanks to diverse sensory feedback (FB). New technologies improve existing techniques or enable new methods to supply the writer with real-time computer-assisted FB. The possibilities are numerous and various. Therefore, two main questions arise: (1) What aspect of the movement is concerned and (2) How can we best inform the writer to help them correct their handwriting? In a first step, we report studies on FB naturally used by the writer. The purpose is to determine which information is carried by each sensory modality, how it is used in handwriting control and how this control changes with practice and learning. In a second step, we report studies on supplementary FB provided to the writer to help them to better control and learn how to write. We suggest that, depending on their contents, certain sensory modalities will be more appropriate than others to assist handwriting motor control. We emphasize particularly the relevance of auditory modality as online supplementary FB on handwriting movements. Using real-time supplementary FB to assist in the handwriting process is probably destined for a brilliant future with the growing availability and rapid development of tablets.

## INTRODUCTION

Handwriting is described as a complex perceptual-motor skill encompassing a blend of visual-motor coordination abilities, motor planning, cognitive, and perceptual skills, as well as tactile and kinesthetic sensitivities (Feder and Majnemer, 2007). Thousands of hours of practice are required to master handwriting skills. Between 12 and 30% of children fail in the motor learning of handwriting (Rubin and Henderson, 1982; Hamstra-Bletz and Blöte, 1993; Smits-Engelsman et al., 2001; Karlsdottir and Stefansson, 2002). These children are considered as poor writers or as having a dysgraphia, namely a learning disability that concerns the mechanical handwriting skill, unrelated to reading or spelling abilities (Hamstra-Bletz and Blöte, 1993). The ability to control graphic movements clearly impacts on the quantity and quality of the written text (Jones and Christensen, 1999). Handwriting control is based on an efficient treatment of feedback (FB). FB is considered here as sensory information that arises from movement (Schmidt and Lee, 2005). Not properly processing the FB generated by handwriting movements could result in poor handwriting and hence impact the academic success of the child.

Since the early 1980s, many studies have investigated the role of sensory FB in the motor control of handwriting (e.g., Smyth and Silvers, 1987; van Galen, 1991; Teasdale et al., 1993; van Galen et al., 1994; Teulings, 1996). Even if no striking change has recently occurred in the way we write that would justify questioning again the sensory signals involved in the perception and control of handwriting movements, the tools we now have at our disposal to study handwriting have dramatically changed and the importance of FB can be reconsidered. Thanks to graphic tablets, we are now able to analyze and closely follow the handwriting process, i.e., the movement generating the trace. Consequently, we can analyze and “dissect” handwriting as a movement *per se*, and not indirectly from the static written trace resulting from this movement. Beyond analysis, it has also become possible to act in real-time on this movement in order to change its control. New technologies have improved existing techniques or enabled new methods of supplying the writer with real-time computer-assisted FB. The possibilities are numerous and various. Therefore, two main questions arise: (1) What aspect of the movement is concerned and (2) How can we best inform the writer to help them to correct their handwriting? These two points, the FB contents and the sensory modality involved, all have to be considered together. Depending on their contents, some sensory media will be more appropriate than others to assist handwriting motor control.

The aim of the present review is to make a synthesis of studies devoted to real-time sensory FB in handwriting in order to evaluate the effectiveness of experimental attempts to improve its learning and rehabilitation. We aim also at envisaging new possibilities. For that, we will firstly report studies on FB naturally used by the writer to control his handwriting. The purpose is to determine which information is naturally carried by each sensory modality, how it is used in handwriting control and how this control changes with practice and learning. Secondly, we will report studies on supplementary FB provided to the writer to help him/her to better control and learn how to write. We will discuss the relevance of each sensory modality according to the information content.

## BASIC SENSORY FEEDBACK IN THE CONTROL OF HANDWRITING

Considering motor skills in general, two modes of control are classically distinguished: a *proactive* control, based on memorized information defined as *internal model* (Wolpert et al., 1995; Wolpert et al., 2011) or *motor program* (van Galen et al., 1994; Schmidt and Lee, 2005) and a *retroactive* control based on sensory FB. Proactive refers to the components of the movement that are anticipated and prepared before the movement is triggered. All these elements are somehow included in the motor command, the role of FB being just to confirm that everything occurs as it was foreseen. Conversely, retroactive refers to all the aspects of the movement which are not programmed before the onset of the movement and which have to be controlled during the ongoing movement on the basis of sensory FB. In addition, sensory FB is also used during learning to improve the planning of the following movement and hence the proactive component. Skilled handwriting in particular involves both types of control: It cannot be totally proactive since several aspects of its production should be controlled in-line. It is considered as semi-automatic. During handwriting learning, Meulenbroek and van Galen (1986, 1988) observed a switch from a proactive (around 5 years) to a retroactive control (around 7 years), followed by a mixed control in older children (around 10 years) when handwriting mastering and motor maturity are reached.

Two types of sensory FB, visual and proprioceptive, are naturally used in handwriting. Two questions arise about them: (1) What are their respective roles and interactions, and (2) How do their roles and interactions change during learning?

### VISUAL FEEDBACK

Visual FB informs about the spatial characteristics of the written trace: where and how the trace is produced. To try to understand the role of vision, many authors have studied the changes of handwriting caused by the absence or the deterioration of visual FB (see **Table 1**).

**Table 1:** Experimental studies on the role of vision in skilled handwriting reported in a chronological order.

Authors	Number of participants	Relevant comparisons	Experimental task	Data analysis	Main results and discussion
Smyth and Silvers (1987)	12 adults	V vs. VC vs. VB vs. NV vs. NVC vs. NVB	Writing of eight sentences	Writing time; Orientation of writing; Writing errors	The orientation of the written words was affected by the loss of vision rather than by the addition of a secondary task. The writing errors were very similar for the secondary task condition and the without-vision condition.
van Galen et al. (1989)	10 adults	V vs. NV	Writing a list of words, with similar vs. dissimiliar upstroke, with and without stroke repetitions within letters, and with long and short letters	MT of correct words	The deprivation of visual FB slowed down handwriting, especially in combination with the conditions that affect the short term memory.
Smyth (1989)	12 adults	V vs. NV	Writing nine letters, nine reverse letters, and drawing eight shapes	Number of strokes; Percentage of occasions on which two start and three progression rules were obeyed in visual and non-visual conditions; Writing errors	Without vision, movement production is simplified to reduce the number of relocations required. The use of consistent directions of movement depends of the ability to use visual control of spatial location.
Burton et al. (1990)	8 adults	V vs. NV in one-fourth, one-half, double, and four-times their normal size	Writing the words ‘poppy’ and ‘wood’	Width of each word; width of the space between the words; width of spaces between letters; width of individual letters; height of ‘tall’ letters (p, y, and d)	Size transformations were greater and closer to the instructed values with than without vision. Variability was greater with than without vision for all three space segments, with no significant effect of vision for any of the three letter segments. With vision, subjects differentiated letters and spaces in making their horizontal transformations; without vision, there was no differentiation.
van Doorn and Keuss (1992)	12 adults	V vs. NV, in the normal vs. short durations	Writing the sequence ‘lenehele’	RT; MT; Size; Spatial variability of the sequence; Variability of MT and Size	Spatial control was not affected by absence of vision but RT increased without vision, suggesting that invariance of shapes is preserved in the absence of vision at the expense of processing time increments.
van Doorn and Keuss (1993)	12 adults	V vs. NV	Writing the sequence ‘lelele’	Size; X/Y -ratio; Spatial variability	Geometric aspects of letters altered under no vision and under the scaling requirement to write in a small format.
Tamada (1995)	8 adults	Visual FB with various delays – 0, 33, 67, 100, 133, 167, 267, and 500 ms	Writing a word	Writing error rate	With increasing the delay, the writing error rate increased, especially in stroke repetition within words. (e.g., “feeling” as “feeeling”). Visual monitoring would be indispensable in producing stroke repetition.
Marquardt et al. (1996)	31 adults	Experiment 1: V vs. VT vs. NV vs. MT	Experiment 1: writing the sentence ‘Die Hunde bellen laut’	Number of Inversion in Velocity per stroke; Size	Vision is not required to produce automated handwriting movements and conscious attention to visual control hampers the elicitation of automated movements. Vision would be used to monitor script size even in highly automated handwriting.
		Experiment 2: VWT in normal size, 133 and 66% of the normal size	Experiment 2: writing ‘ll’ repeatedly for 8 s		

*V, writing with vision; VC, with vision while counting; VB, with vision while saying ‘blah’; NV, writing with no vision; NVC, with no vision and counting; NVB, no vision and ‘blah’; VT, visual tracking of the pen; MT, mental tracking; VWT, vision of the written trace only; MT, movement time; RT, reaction time.*

Two types of constraints have to be dealt with in handwriting production: those related to the formation of the letter’s shape and those related to the spatial layout and sequencing of the letters on the paper. Paillard (1990) referred to these two types of spatial constraints as the ‘morphocinetic’ and the ‘topocinetic,’ respectively. The morphocinetic component of handwriting concerns the shape of the letters (the “*what?”* in handwriting). In skilled handwriting, the shape of the letters is perfectly known and mastered; therefore the morphocinetic component is almost independent of visual control. Alternatively, the topocinetic component, which concerns the spatial layout of the text in the graphic space, the spacing between letters and words, the placement of punctuation, etc. (the “*where?”* in handwriting) requires visual FB. The two components are not identically controlled by visual FB: in the case of experts, suppressing visual FB mainly affects the topocinetic component, without compromising the morphocinetic one (Paillard, 1990). Nevertheless, when letters are complex and composed of several strokes, they are produced under visual control. As a matter of fact, a decrease in the number of strokes and an alteration of the sequence and direction of movements has been shown when writers did not use visual information (Smyth and Silvers, 1987; Smyth, 1989;). Visual FB would update information concerning letters with repetitive strokes in the motor buffer memory (van Galen et al., 1989; van Galen, 1991). In the same vein, Tamada (1995) investigated the effects of delayed visual FB on the handwriting of some familiar words, using various delays between 0 and 500 ms. She observed that writing errors increased with the delay, with a tendency for some additional strokes to be inserted, especially for letters with repetitive strokes, which is in accordance with the model of van Galen et al. (1989).

The handwriting perturbations induced by the absence of vision revealed the crucial role vision plays in the control of the written trace. Nevertheless, expert writers are able to minimize the impact of vision suppression by developing adaptive strategies. This conclusion was drawn by van Doorn and Keuss (1992) by evaluating the movement time and the reaction time in handwriting both with and without vision. They showed that preserving the morphocinetic component required more time when unseen. They confirmed different strategies in the handwriting production without vision to maintain, to a certain level, a spatial invariance (van Doorn and Keuss, 1993). According to another hypothesis, increasing proprioceptive FB during execution could compensated for the absence of vision. This hypothesis was strengthened by the observation that an increase of pressure and size occurred in the absence of vision, as if the writers tried to maximize their proprioceptive perception (van Doorn, 1992).

Consequently, there is general agreement that writing without vision changes the written trace, particularly the topocinetic components. But, does it also affect the kinematics of the handwriting movement? Marquardt et al. (1996, 1999) analyzed the velocity profile both with and without visual FB. When writing both with and without vision, all subjects produced a smooth and single-peak velocity profile. In a second experiment, the authors manipulated the visual control of the written trace, at normal size, 133 and 66% of the normal size. They confirmed that visual FB is not required to produce and control automated handwriting movement but rather to take into account the spatial constraints of the trace (e.g., the size etc.), which seems to hamper the elicitation of automated movement, a conclusion already drawn by Burton et al. (1990).

In conclusion, vision plays a crucial role in the control of the written trace and allows the writer to correctly link words, letters, and strokes within letters (those with repetitive strokes). However, the absence of vision does not significantly affect the handwriting process, i.e., the ongoing movement that generates the trace. Quite the reverse, suppressing vision while writing would promote a more proactive control that would help the “blind” writer to write fluently, at the risk of being less accurate and, consequently, less legible.

### PROPRIOCEPTIVE FEEDBACK

Proprioceptive FB arises from muscles, tendons and joints receptors and informs about the positions and movements of limbs. In principle, tactile perception conveyed by cutaneous receptors is not directly included in proprioception. However, for the sake of simplicity we will not consider it separately from proprioception, particularly because when deafferented patients lose proprioception, they also lose tactile perception. Kinesthesia refers to sensory information about movements, not including static positions: therefore, kinesthesia and proprioception do not totally overlap. In handwriting, kinesthetic FB can inform about spatial, kinematic, and/or dynamic characteristics of handwriting movement whereas tactile FB from skin receptors can inform about the pressure exerted by the fingers onto the pen and thus about the forces exerted during handwriting. Of course, removing proprioception is not as easy as closing the eyes, therefore quantifying how far a writer takes into account proprioceptive FB is difficult. This is why only a few studies have been conducted and they only investigated the absence of proprioceptive information in study-cases of deafferented patients (e.g., Ghez et al., 1990; Teasdale et al., 1993; Hepp-Reymond et al., 2009).

Teasdale et al. (1993) asked a deafferented patient to write with and without vision. The comparison of the trace written by the patient using vision and that of the control subjects did not reveal a difference attributable to the lack of proprioception. However, when the patient’s handwriting with and without vision was compared, only the topocinetic component was affected by the absence of vision, i.e., when she was lacking all FB. In other words, the absence of proprioceptive FB did not affect the written trace: The morphocinetic component (form of the letters) was preserved and only the topocinetic component deteriorated due to the absence of both vision and proprioception. These findings confirm that, in skilled handwriting, proprioceptive FB is not fundamental for controlling either the shape of the letters or their spatial layout on the page: the former would be controlled by a proactive mode and the latter by visual FB.

If proprioception does not inform about the “product” of handwriting (the written trace), does it in form about the process (the movement)? To try to answer this question, Hepp-Reymond et al. (2009) quantified precisely the role of proprioception and vision in a deafferented patient and healthy participants. They compared 13 handwriting variables in the cursive writing of a word. Where Teasdale et al. (1993) concluded that proprioception played a weak role in handwriting trace, Hepp-Reymond et al. (2009) showed that handwriting movement was clearly affected by the lack of proprioception. More precisely, they demonstrated that three variables (number of pen lifts, number of inversions in velocity profile, and mean stroke frequency) changed without proprioceptive FB, whatever the visual conditions. In the patient who lacked proprioception, the written words remained legible provided she benefited from visual FB, but the movement was affected.

Whether the movement deterioration only results from a lack of kinesthetic FB, from a lack of tactile FB or from both, remains an open question. Only one study (Ebied et al., 2004) was devoted to the lack of tactile FB in healthy participants (by infiltrating a local anesthetic around the median nerve at the wrist). The authors observed that blocking cutaneous sensation did impair the ability to write, as judged by an increase in the movement time and in acceleration fluctuations. These findings highlight the importance of touch in handwriting control, though more studies are necessary to provide clear conclusions about its specific role. The number of studies devoted to the role of cutaneous FB is probably going to increase in the future, with the rapid development of smartphones and tactile tablets in which the pen tends to be replaced by the finger (e.g., Tu and Ren, 2013).

In conclusion, proprioceptive FB does not really seem to inform about the spatial characteristics of the written trace, but it is useful for controlling the kinematics and dynamics of handwriting movement. Therefore, suppressing proprioceptive FB has the opposite effect of suppressing visual FB, confirming that vision and proprioception are clearly complementary in handwriting control.

What does happen when visual and proprioceptive FB are not congruent? Changing the congruence of the visual FB, for instance in a mirror-drawing task, induces a conflict between visual and proprioceptive FB. This conflict permits the study of the relative contributions of the two sensory modalities. Lajoie et al. (1992) demonstrated that a deafferented patient had no problem achieving a mirror-drawing task, whereas healthy participants needed more than four trials to attain a similar performance. They proposed that the inversion of visual coordinates imposes a recalibration because of the conflict with proprioceptive FB. In the deafferented patient, the conflict does not exist. Using the same mirror-drawing protocol in healthy subjects, Gullaud-Toussaint and Vinter (1996, 2003) observed the dominance of either the visual or the proprioceptive modality within the visual-proprioceptive conflict. They distinguished two strategies, one favoring vision which preserved the perceived movement directions, but in turn induced a reversal of the directions drawn on the sheet of paper, the other favoring biomechanical constraints which tended to preserve the directions drawn on the sheet of paper, but in turn provoked a reversal of the directions perceived in the mirror condition.

### HOW DO VISUAL AND PROPRIOCEPTIVE FEEDBACK CHANGE WITH LEARNING AND DEVELOPMENT?

Mastering handwriting requires several years of practice usually achieved during childhood, therefore, handwriting motor control, and hence the use of FB, change both with the increase in learning and development of the brain and body (e.g., Chiappedi et al., 2012). Handwriting movement control evolves from a retroactive to a more proactive mode with learning (Schmidt and Lee, 2005). However, the change in handwriting control is not monotonic during the child’s development: it evolves from an initial predominance of fast ballistic movements at 5–6 years to the mature medium-speed ballistic movements, around 9–10 years, via a relatively unstable period at 7–8 years (Meulenbroek and van Galen, 1986, 1988).

Meulenbroek and van Galen (1986, 1988) interpreted these changes as a switch from a proactive (around 5 years) to a retroactive control (around 7 years), followed by a mixed control in older children (around 10 years). Asking 6 to 9 year-old children to increase their execution speed improved the fluency. Since increasing the speed reduces the time available to take into account the FB, they concluded that it would induce a more proactive mode of control. In the same vein, Chartrel and Vinter (2008) studied the effect of temporal (speed) and spatial (size) constraints on cursive letter production in 5 to 7 year-old children. The idea was that temporal constraints decrease visual control and hence improve fluency. They observed the same effect of spatio-temporal constraint at the age of 6 and 7 years, but not at 5 years. In line with the study by Hay (1984), they suggested that 5 year-old children control their movement more proactively. In conclusion, additional spatial cues may help visual FB in young children and speed constraint would be more beneficial to older children.

In addition to a global reduction of FB use, increasing expertise in motor control can be explained by a gradual change in the balance between visual and kinesthetic control (Fleishman and Rich, 1963; Schmidt and Lee, 2005). The underlying argument was that at the beginning of learning, the learners do not have a kinesthetic reference of the movement and hence control it visually. With practice, this kinesthetic information is memorized and then used as reference for executing the following movements, thus reducing the need for visual control. Laszlo and Bairstow (1984) aimed at linking handwriting performance in 5 to 6 year-old children with kinesthetic sensibility. They showed that children who, following specific training, had improved their kinesthetic sensitivity, had also improved their handwriting skills. They concluded that the lack of kinesthetic readiness, a term proposed by these authors, explains the difficulty that may hinder effective training of writing at this age. They suggested delaying formal training of handwriting until the age of seven, when most children develop kinesthetic readiness naturally. However, examining the effect of kinesthetic training on the handwriting performance in first graders, Sudsawad et al. (2002) did not successfully link kinesthesia and handwriting. The positive effect of kinematic training on handwriting remains unclear and should be considered with caution.

Does visual FB have the same importance in children who are learning to write and in adults mastering their handwriting? We previously mentioned that only the spatial organization of the written trace was affected by the absence of visual FB in adults: not the movement kinematics. Chartrel and Vinter (2006) compared the role of visual FB in 8 to 10 year-old children and they showed that the absence of visual FB was more detrimental in younger (8 year-old) than in older (10 year-old) children and adults. Without vision, movement time increased and movement fluency decreased in the youngest children whereas the handwriting kinematics was not changed by absence of visual FB in adults. To conclude, visual FB would be crucial in children who are beginning to learn how to write and who have not yet a complete representation of the shape of letters. In addition, the younger children may not be able to process the proprioceptive signals about movement dynamics. Therefore, suppressing the visual FB in children would probably lead them to write differently, for instance at a larger size, resorting more to proprioceptive signals, but without favoring the automatization of movement.

### CONCLUSION

Two sensory modalities are naturally used for controlling handwriting: vision and proprioception. Visual FB is used mainly for controlling the spatial layout of the written trace. Proprioceptive FB is used for controlling movement execution, thus freeing vision for other controls. The lack of proprioceptive FB does not affect the capability of writing a legible trace thanks to visual control. When visual and proprioceptive FB are not congruent, a conflict appears leading to a sensory adaptation, which seems to be differently handled according to individual preferences and strategies and to the expertise level. During the learning process, visual control is used significantly at the beginning, but gradually decreases making way for a more automatic control and a change in the balance between visual and proprioceptive control. If this is the case, a possible strategy for the optimization of handwriting learning or the rehabilitation of handwriting troubles would be to facilitate the switch from control based on the written trace to control based on the movement, in order to write both precisely and fluently. The question is how supplementary FB may help with that.

## SUPPLEMENTARY SENSORY FEEDBACK

Supplementary FB refers to additional sensory information provided to a writer, in complement to, or in compensation for, natural sensory FB. Therefore, supplementary FB can have two practical benefits: it can facilitate handwriting rehabilitation; it can help handwriting learning in children. We assume that providing supplementary FB to a proficient writer has little or no interest. Indeed, the *Optimal feedback control* model (for a review, see Todorov, 2004) suggests that the central nervous system sets up FB controllers that continuously convert sensory inputs into motor outputs, and that these are optimally tuned to the goals of the task by trading off energy consumption with accuracy constraints. Consequently, corrections of task-irrelevant errors are not only wasteful but they can also generate task-relevant errors (Wolpert et al., 2011).

In the usual protocols of handwriting rehabilitation, therapists correct handwriting mainly by examining the written trace. This is similar to what is done in the *Motor control* domain, when supplementary FB based on the Knowledge of the Results (KR) is supplied to the subject (Schmidt and Lee, 2005). Another possibility is to provide supplementary FB about the handwriting movement itself. This method is referred to as Knowledge of Performance (KP). Contrary to KR, which is provided after the performance of an action, KP can be provided either after or during the performance. Note that sometimes therapists give on-line FB, when they give a verbal comment about the velocity of the arm or when they hold the writer’s hand to mimic the expected movement in order to make them feel the correct movement. In the present review, we will only discuss the latter option: supplying writer with real-time supplementary FB during the execution of handwriting movement in order to improve their control and aid in their rehabilitation.

Supplementary real-time FB makes it possible to change or provide additional information that a writer can access, or to supply the writer with information not naturally accessible. In other words, it can be used to amplify existing FB or give new information, not directly supplied by this FB. Although using supplementary FB to improve handwriting movements is not a recent idea (e.g., Søvik and Teulings, 1983), only recently have the increasing capacities of writing tools (e.g., graphic tablets or haptic devices) and of computers made it possible to receive real-time computer-assisted sensory FB. These new types of FB can concern the two sensory modalities already used in basic FB, vision and proprioception, and another sensory modality, audition, not naturally involved in handwriting.

### SUPPLEMENTARY VISUAL FEEDBACK

Real-time visual FB has seldom been tested in handwriting because it has several limits. First, adding supplementary visual information, in a task where vision is already used to control the trace, increases the difficulty for the writer. It requires some sharing of attention that may be detrimental, especially at the beginning of learning. This was, for instance, a criticism leveled at lined paper, which adds a supplementary visual element that beginners have to cope with in addition to forming the letters they are tracing. Such a supplementary visual cue might actually compromise legibility (Weil and Amundson, 1994). Secondly, perceiving and processing visual information requires too much time to be compatible with the fast corrections that occur during handwriting (Teulings and Schomaker, 1993). Consequently, supplementary visual FB tends to slow down handwriting and to make it dysfluent, as demonstrated by Portier and van Galen (1992). Thirdly, supplementary visual cues might modify the nature of the task, transforming it into a copy task not requiring the same cognitive processes (Gonzalez et al., 2011). Finally, as already mentioned, vision is very informative concerning the spatial features of handwriting (the correctness of the letters, their position on the paper and position relative to each other…). However, vision is not the best sensory modality for informing optimally about the dynamic features of handwriting.

Nevertheless, if therapists do really want to add visual FB, we would advance three possibilities: First, this FB should be displayed after and not during the ongoing movement (Portier and van Galen, 1992). Supplementary visual FB on the velocity and the smoothness of the movement has been shown to be efficient when distributed after the movement (Søvik, 1981; Søvik and Teulings, 1983). The results showed that this supplementary FB improved the writing speed without diminishing accuracy, but the smoothness did not change significantly, probably because of methodological reasons. Indeed, the smoothness index was computed on the basis of the absolute velocity variability. The normal fluctuations of absolute velocity, resulting from fluctuations of curvature in the trajectory formation (Viviani and Terzuolo, 1982; Lacquaniti et al., 1983), were not taken into account.

Another possibility could be to profit from the variety of information conveyed by vision, for instance by changing in real-time the color of the ink according to a given kinematic variable (e.g., velocity or fluency). This is easy to do, from a technical point of view, thanks to graphic tablets equipped with a tactile screen. One should, however, ensure that the kinematics do not differ too much between paper and tactile screen surfaces. We would not recommend using a digitalized tablet connected to an external screen for two reasons: first, such a procedure implies that the visual FB and the proprioceptive feeback do not coincide anymore in space; secondly the change from a horizontal to a vertical plan for visual FB requires a visuomotor adaptation (grossly equivalent to a mental rotation). Both changes may affect handwriting control, especially in children with learning difficulties.

Finally, instead of adding supplementary information, another possibility could be to partially reduce visual FB. For example, with a graphic tablet it is possible to suppress the visual trace (but to preserve the vision of the pen and of the useful spatial cues like the position on the page, the line break…) and thus to let the writer focus on their movement. This could be a good way to prevent the writer from paying exclusive attention to the visual trace.

### SUPPLEMENTARY PROPRIOCEPTIVE FEEDBACK

Applying supplementary proprioceptive FB may be the most intuitive way of helping the writer to perceive the correct movement. This is to some extent what teachers do when they hold a child’s hand and drive it along the correct trajectory in order to make him/her produce and perceive the correct movement. Applying proprioceptive FB requires the use of a mechanical device able to guide the hand of the writer: hence the generic term of *haptic guidance*. The haptic devices used in handwriting were usually multi-jointed robot arms that produce forces and allow a positioning of the pen anywhere in its workspace (see for instance Bluteau et al., 2008). A strong requirement of this method is the necessity of an *a priori* model of the ideal trajectory that the writer should reproduce. Thanks to force FB devices, haptic guidance not only informs on the current movement but also actively corrects it in relation to positional, kinematic or force errors. The question is thus to identify what the best haptic guidance is between a correction based on spatial error, focusing on the correct shape, and a correction based on force error, focusing on the correct movement.

The first studies devoted to haptic guidance were conducted on Japanese (Henmi and Yoshikawa, 1998; Yoshikawa and Henmi, 2000) and Chinese handwriting learning (Teo et al., 2002). Although they were promising, the results of these first attempts were not supported by any statistical analyses. Moreover, the learner was passively driven by the robot along the correct trajectory previously recorded by the teacher. The learner had no latitude to wander from this imposed trajectory. Therefore the FB was imposed on the passive writer. Thanks to the *Reactive Robots System* (Solis et al., 2002), the possibility appeared of taking the writer’s performance into account by modifying the force FB in real-time. The Reactive Robots System not only reproduced the model, but also identified the character initiated by the writer among a panel of memorized characters and then adapted the force FB to the trajectory corresponding to the identified character. Again, the effect of this tool was not evaluated under strict experimental conditions and criteria. In particular, a comparison with a control group who did not benefit from the device was not made and therefore drawing any conclusion about the effectiveness of this method is difficult.

More recently, haptic guidance was evaluated under more rigorous experimental conditions for handwriting learning (Palluel-Germain et al., 2007). The authors evaluated the effect of a visuo-haptic device in 5 to 6 year-old children carrying out a copying task. Kinesthetic FB was based on positional error, by comparing the produced trajectory and the nearest point of the model trajectory. They found that children who learned with the visuo-haptic device exhibited more fluent movements. Interestingly, they concluded that augmented kinesthetic FB in such a learning protocol increased the proactive strategy of the child’s control. One year later, Bluteau et al. (2008) tested the efficiency of two kinds of haptic guidance in adults, based on position or force errors. The position error corresponded to the Euclidean distance between the ideal trajectory required by the task and the produced trajectory. The force error corresponded to the difference between the force produced by the user and the force used in the model guiding the robot for the theoretical trajectory. These last authors got a positive effect of haptic guidance based on force error alone.

In conclusion, applying haptic guidance for changing proprioceptive FB seems relatively promising, provided that the guidance is based on a dynamic error and not on spatial error. However, its effectiveness remains to be confirmed because this device has been evaluated mostly on simple motor tasks (for a review, see Sigrist et al., 2013), but to a lesser extent on more complex tasks like handwriting. In addition, haptic guidance necessitates specific devices that can be costly and complex to use. Furthermore, as already explained, proprioceptive guidance with force FB devices requires an initial recording of an ‘ideal’ trajectory that the writer would then have to reproduce and from which corrections could be made. The individuality and variability of poor handwriting brings into question the validity of methods based on dynamic model reproduction for rehabilitation. This question has not been addressed until now.

### SUPPLEMENTARY AUDITORY FEEDBACK

Sounds can be used to add supplementary information that the writer does not take into account or that he/she cannot access naturally (e.g., inform about the muscular activity, Ince et al., 1986). It can also be used as an alternative channel of information processing in order to compensate for a deficit in another sensory modality (e.g., compensate for a visual deficit by giving spatial information, Plimmer et al., 2011). Until now, no study has investigated the role of audition in handwriting motor control. Handwriting has always been considered as a silent activity. Because there is no link *a priori* between handwriting and sounds, auditory FB could be used to inform on many different variables. The question is thus to discover how and on what, sounds may help in improving motor control or the relearning of handwriting.

Historically, auditory FB in handwriting has only been used to treat one particular neurological deficit: namely writer’s cramp. Reavley (1975) was the first who tried to transform EMG activity into sounds in order to supply patients with auditory biofeedback. He wanted to help them to better contract muscles that were not appropriately activated. The author reported improvements in terms of “quick, effective and legible handwriting”. However, he did not describe the apparatus and auditory FB used. Other attempts to inform patients about their muscular activity were made with auditory FB varying in intensity as a function of EMG activity. Bindman and Tibbetts (1977) treated six patients over periods ranging from 3 months to 5 years. Auditory FB consisted of increasing or decreasing sound intensity, depending upon muscle contraction or relaxation. The authors reported that one patient became completely symptom-free, one improved sufficiently to produce little or no disability at work, two improved but continued to have some work disability and two evidenced no change. However, as reported by Ince et al. (1986), no details were provided in this article on any aspect of methodology, apparatus, muscle activity, or data analysis. No pre- or post-treatment handwriting samples were included for visual inspection of the changes. In another study (Cottraux et al., 1983) the auditory biofeedback consisted of an analog audio signal that the patients had to reduce in pitch by decreasing the tension of their muscles. Again, no statistical analyses were performed and several methodological criticisms can be raised (Ince et al., 1986 for more details). O’Neill et al. (1996) reported a case of a patient whose symptoms disappeared after one week of such treatment. Such rapid effects were surprising and Deepak and Behari (1999) made another attempt on ten patients with Writer’s Cramp and hand dystonia. They revealed that nine of them showed an improvement of between 37 and 93% in handwriting after daily practice with auditory biofeedback over a few months. Nevertheless, although the technique seemed relevant, the biofeedback from EMG activity was questioned by Ince et al. (1986) and by Deepak and Behari (1999) who admitted that the first results were not totally convincing. One of the limitations was that handwriting involves many muscles, sometimes small and profoundly located, whose activity is difficult to record. Possibly, more invasive methods (e.g., intra-muscular EMG) would overcome this problem, but such methods remain difficult to use and restricted to serious neuromuscular pathologies.

Muscular activity is directly linked to forces exerted by the muscles, and patients suffering from writer’s cramp are known to apply too great a force with their fingers onto the pen. Therefore, transforming the grip force into sounds has been tested as a way of leading patients to decrease the force of their grip (Baur et al., 2009). The pen was equipped with force sensors and the auditory FB consisted of a continuous low-frequency tone when the average grip force exceeded 5 N. The tone frequency increased in four steps with the grip force level and patients were instructed to perform the writing exercises in such a way that they heard a pleasant, low-frequency tone. After several hours of training, the authors reported that both the grip force and the vertical pressure applied by the pen on the paper decreased in the patients. This easy to apply and non-invasive method seems quite encouraging for the rehabilitation of writer’s cramp.

In addition to a simple association between a physiological variable and a sound, such as those previously described, more complex associations can also be used when the goal is to supply auditory FB about movements: This is the so-called movement sonification (Effenberg, 2005; see Sigrist et al., 2013 for a review). In the case of handwriting, the purpose is to enrich perception by adding auditory signals linked to given variables of handwriting movement (spatial, kinematic, or dynamic information). According to the values of the chosen variables, one or several sound parameters can be modified. As Sigrist et al. (2013) observed, however, it is fundamental to define as precisely as possible the sonification strategy. Two issues have to be solved: the ‘*what to sonify?’* consists of identifying precisely which variables should be sonified, i.e., the sound mapping; the ‘*how to sonify’* consists of evaluating the auditory design. Now, with regards to handwriting, there is a lack of solutions to these problems in the current literature.

Is auditory FB relevant for informing about the spatial characteristics of handwriting? Andersen and Zhai (2010) made a first attempt at answering this question. They compared both the accuracy of the written trace and the speed of execution under four conditions resulting from the crossing of two types of FB: with and without visual FB on the written trace, and with and without supplementary auditory FB linked to the pen’s position. They related a positive effect from supplementary auditory FB on the motivation of the learners, but no direct influence on their performance. This absence of effect can be explained by the fact that spatial information is harder to translate into the auditory than into the visual dimension (Welch, 1999).

On the other hand, the dynamic features of sounds make them particularly appropriate for signaling the movement’s dynamics. Indeed, when listening carefully to the noise produced by handwriting, one can hear a friction sound generated by the pen–paper interaction, especially when the surface is rough. This friction between the pen tip and the paper’s asperities produces sound variations related to the handwriting kinematics that may, to a certain extent, inform on what the writer is writing. Thoret et al. (2014) tested this hypothesis with a synthetic friction sound whose timbre variation was related to the pen’s velocity. In a first task, they demonstrated that the timbre variations produced from the sound of a moving pen appear to vary in accordance with the kinematic rule governing real graphical movements. In a second experiment, the authors investigated the ability to categorize drawn shapes ‘by ear’. Subjects were asked to associate friction sounds with simple graphic shapes. They concluded that categorization of visual shapes on the basis of their produced sounds was possible if the kinematics differ sufficiently. However, these results were acquired using simple graphic shapes that had been drawn by the very fluid movements of an adult writer. Contrary to drawing simple shapes, handwriting imposes complex movements which are not always fluid, in particular for handwriting learning or rehabilitation.

(Danna et al., 2013a,b, 2014) have studied the effect of movement sonification for handwriting learning and rehabilitation. The sonification strategy consisted of using an intuitive mapping between sound and movement, i.e., friction sounds that might have been naturally created by real pen movements. The timbre of the sounds varied with the instantaneous velocity of the pen (see Thoret et al., 2014). To evaluate the potential of the technique, these authors carried out a series of experiments. The first experiment was designed to answer the question: “Is it possible to identify poor handwriting only by ear, without seeing the written trace?” (Danna et al., 2013a). In a pre-experiment, samples of the same word written on a graphic tablet by children with dysgraphia, children with proficient handwriting, and proficient adult writers were collected. Then, from these samples, three handwriting variables – the instantaneous velocity, the movement fluency, and the axial pen pressure – were sonified in order to create audio files which were then played to naïve adult listeners who had to mark the quality of the underlying unseen handwriting. The listeners were not aware that the sounds corresponded to three different groups of writers. The results showed that, when they were informed about the meaning of the sounds and the evaluation criteria, all listeners marked the dysgraphic handwriting lower than that of the two other groups. So it appeared possible to discriminate only by ear between proficient and poor handwriting. This result validated the sounds used for informing on the quality of handwriting. However, the sounds here were not FB: they were not played back to the person whose handwriting movements had generated them.

The same sonification strategy was applied as real-time auditory FB for improving handwriting learning (Danna et al., 2014) and rehabilitation (Danna et al., 2013b). Auditory FB improved the learning of new characters in adults with their non-dominant hand in a single training session (Danna et al., 2014). A positive effect was also obtained in a rehabilitation protocol lasting several weeks involving children with dysgraphia (Danna et al., 2013b). However, the performance increase with sonification should be compared to a control situation where children do not benefit of the sonification, and this was not done in this pilot study.

In conclusion, applying concurrent auditory FB seems very promising, provided that the auditory FB informs about handwriting movement and not about a spatial characteristics. Indeed, vision is more appropriate than audition for perceiving spatial information, whereas sounds can naturally reveal phenomena containing dynamic cues to which the eye is less sensitive (e.g., Fitch and Kramer, 1994). Moreover, auditory FB may be efficient without leading the learner to become dependent on external FB. Ronsse et al. (2011) demonstrated that learners are less dependent on auditory than on visually augmented FB. Because audition is available during handwriting, sounds may be used to complement visual and proprioceptive FB and enlighten the writer about “hidden” dynamic variables which are not sufficiently taken into account, particularly at the beginning of learning or in rehabilitation. However, the efficiency of auditory FB depends considerably on its correct interpretation, since listening to auditory displays is less common than viewing visual displays (Sigrist et al., 2013). Finally, in addition to their informative characteristics, sounds can be fun and can motivate learners. Since handwriting learning or rehabilitation requires daily training over several months, the learner’s motivation is one of the most important components to take into account.

Another possibility consists of applying multimodal concurrent FB. To our knowledge, only one study has proposed a multimodal system based on coupled auditory and haptic FB to help blind children to sign (Plimmer et al., 2011). They showed that with such multisensory FB, blind children more quickly learned to sign. No precise information regarding the kinematic variables was reported in this clinical study.

## CONCLUSION

The mastering of handwriting is so essential in our society that it is important to try to find new methods for facilitating its learning and rehabilitation. With the technical means we now have at our disposal, supplying writers with new types of sensory FB that are richer than those naturally used, is easily conceivable. We have just recalled that two sensory modalities are involved in handwriting control: vision and proprioception, and they do not inform on the same aspects of handwriting. Vision is more suited for checking the quality of the written trace and proprioception for controlling the ongoing movement. Providing enriched FB in each of them is theoretically possible, however, these types of FB should respect their specificities. In particular, a new visual type of FB can hardly be provided during the execution of the movement without the risk of overloading the cognitive capacities and inducing a subsequent degradation of the movement. Proprioceptive supplementary FB is likely to be more appropriate to facilitate the execution of a fluent movement, however it can be costly and is not easy to use. Finally, another sensory modality, namely audition, which does not naturally contribute to handwriting control, could be a good candidate for adding supplementary FB. Audition has several advantages: first, supplementary auditory FB is less likely to overload the cognitive process than additional visual FB, second it is particularly suited to informing about the unfolding of a process and hence about the movement kinematics, third it is easy to use, and finally it might add an element of play to the learning process, particularly for children. Enriched handwriting based on new multisensory FB is also conceivable. Using real-time supplementary FB to assist in the handwriting process is probably destined for a brilliant future with the growing availability and rapid development of tablets.

## Conflict of Interest Statement

The authors declare that the research was conducted in the absence of any commercial or financial relationships that could be construed as a potential conflict of interest.
